# Multiple receptor conversions during the course of metastatic breast cancer therapy: a case report and review of the literature

**DOI:** 10.1186/s13256-022-03679-y

**Published:** 2022-12-13

**Authors:** Yolcar Chamorro, Ana C. Sandoval-Leon, Charles L. Vogel, Michaela T. Nguyen, Grace Wang

**Affiliations:** 1 Miami Cancer Institute/Baptist Health South Florida, 8900 North Kendall Drive, Miami, FL 33176 USA; 2 Miami Cancer Institute/Baptist Health South Florida Plantation Branch, 1228 South Pine Island Road Suite 410, Plantation, FL 33324 USA

**Keywords:** Breast cancer, Receptor conversion, Discordance, Circulating tumor DNA, Biopsy, Heterogeneity, Case report

## Abstract

**Background:**

Adjuvant systemic therapy decreases recurrence and death from breast cancer, but late relapse still occurs. Therapeutic decisions are based heavily on receptor tissue characterization. Even though the vast majority of metastatic sites do not have receptor conversions, they can occur at the time of metastasis and/or during the course of treatment. However, multiple receptor conversions are uncommon.

**Case presentation:**

We present an unusual case of a Caucasian patient originally diagnosed with an estrogen receptor-positive, progesterone receptor-positive, and human epidermal growth factor receptor 2-negative primary breast cancer who had a recurrence after 15 years of therapy. Her metastatic tumor had a different receptor status than the original tumor. During the course of therapy, at the time of progression, a new biopsy showed that her tumor had changed once more.

**Conclusion/Discussion:**

Tracking receptor conversions is important in metastatic breast cancer treatment. Single receptor conversions have been documented to occur, but not much is known of multiple receptor conversions. This case sheds light on the possibility of patients having multiple receptor conversions and the importance of performing multiple biopsies during the course of metastatic treatment.

## Background

Breast cancer (BC) is the most common cancer affecting women in the USA, and the second leading cause of cancer-related deaths in women after lung cancer [1]. The American Cancer Society estimates that 287,850 new cases of invasive breast cancer will be diagnosed in 2022 among American women [1]. Estrogen receptor (ER) positive, human epidermal growth factor receptor 2 (HER2) negative is the most common type of BC, accounting for 65% of cases in women less than 50 years old and 75% of cases for older women [2]. The use of endocrine therapy with tamoxifen or aromatase inhibitors for 5–10 years is highly effective in reducing recurrence rate and death, yet endocrine resistance and late relapse may still occur [3–5]. Pan *et al*. conducted a meta-analysis that showed that BC recurrences steadily occurred 20 years after the original diagnosis in patients that completed 5 years of adjuvant endocrine therapy [6]. Furthermore, metastatic BC (MBC) was strongly linked to the original tumor diameter and nodal status (TN), with recurrence risks ranging from 10% to 41% depending on tumor grade and TN status [6]. Although strides have been made in treating BC in recent years, MBC is still considered to be essentially incurable. It is of interest, however, that in the CLEOPATRA trial, a small subset of HER2 MBC patients that remain progression free for 8 years are believed by some observers to effectively be cured by the use of pertuzumab, trastuzumab, and docetaxel [7].

The status of ER, progesterone receptor (PR), and HER2 are vital in guiding clinicians in their therapeutic decisions. In the past, these decisions were largely based on tissue characterization of the primary site and not always the metastatic sites. However, it has been shown that changes in ER, PR, and HER2 status from the primary breast tumor to distant metastatic sites do occur, affecting the choice of systemic therapy. These receptor conversions are hypothesized to be the result of tumor heterogeneity and clonal selection as a result of treatment [8, 9]. To our knowledge, there is limited information on multiple receptor conversions in one single patient.

Herein we present a case of a patient who had changes in her receptor status twice during the course of her disease, and how repeating a biopsy at the time of disease progression was key to determining her therapy.

## Case presentation

A 49-year-old Caucasian woman was originally diagnosed with an ER-positive (3+), PR-positive (1–2+), HER2 (2+), fluorescence in situ hybridization (FISH)-negative cancer in 2003. It was a grade 3, T2N2M0, left-breast infiltrating ductal carcinoma. *BRCA1* and *2* analyses were negative. She was treated with four cycles of neoadjuvant docetaxel, doxorubicin, and cyclophosphamide (TAC) and subsequently had a left mastectomy with axillary lymph node dissection. Repeat biomarkers showed that it was ER positive (3+), PR negative, and HER2 (2+), negative by FISH. The patient completed adjuvant radiation therapy. She then received adjuvant tamoxifen for 2 years followed by aromatase inhibitors continuing for 13 years until recurrence.

**Fig. 1:**
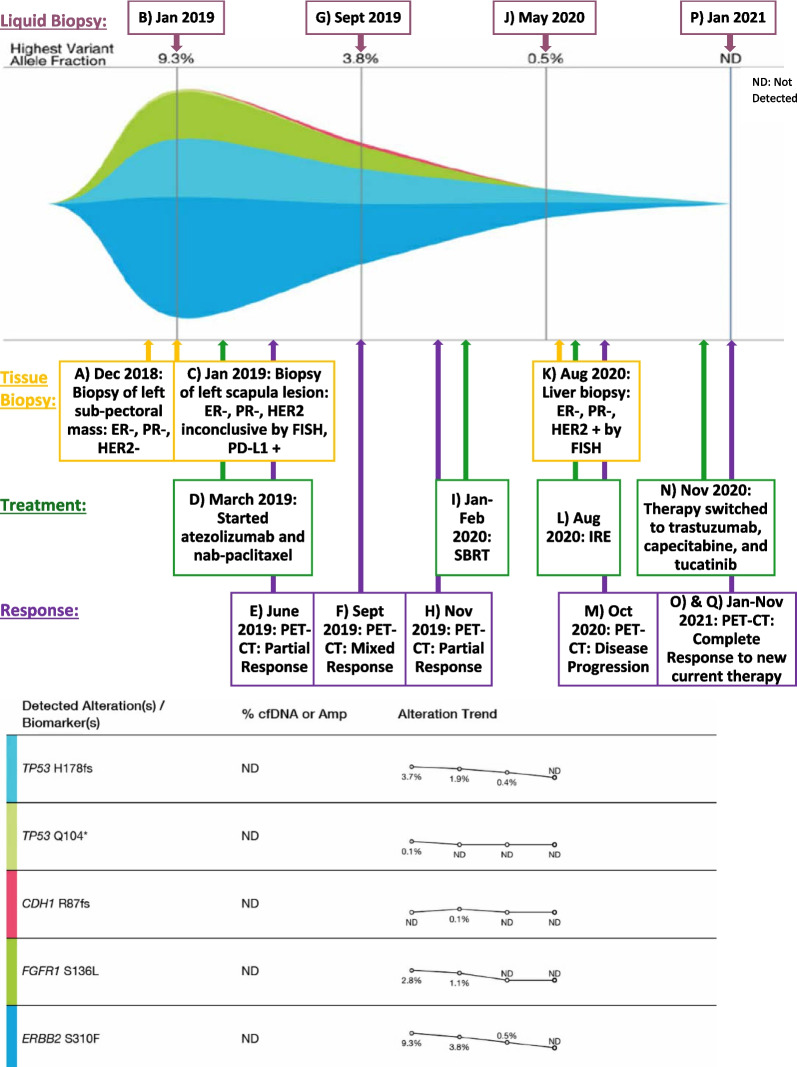
Timeline and treatment history after metastasis with circulating tumor DNA in a tumor response map (the map and table below illustrates the variant allele fraction detected of the observed somatic variants during each sample submission of liquid biopsy)

**Fig. 2:**
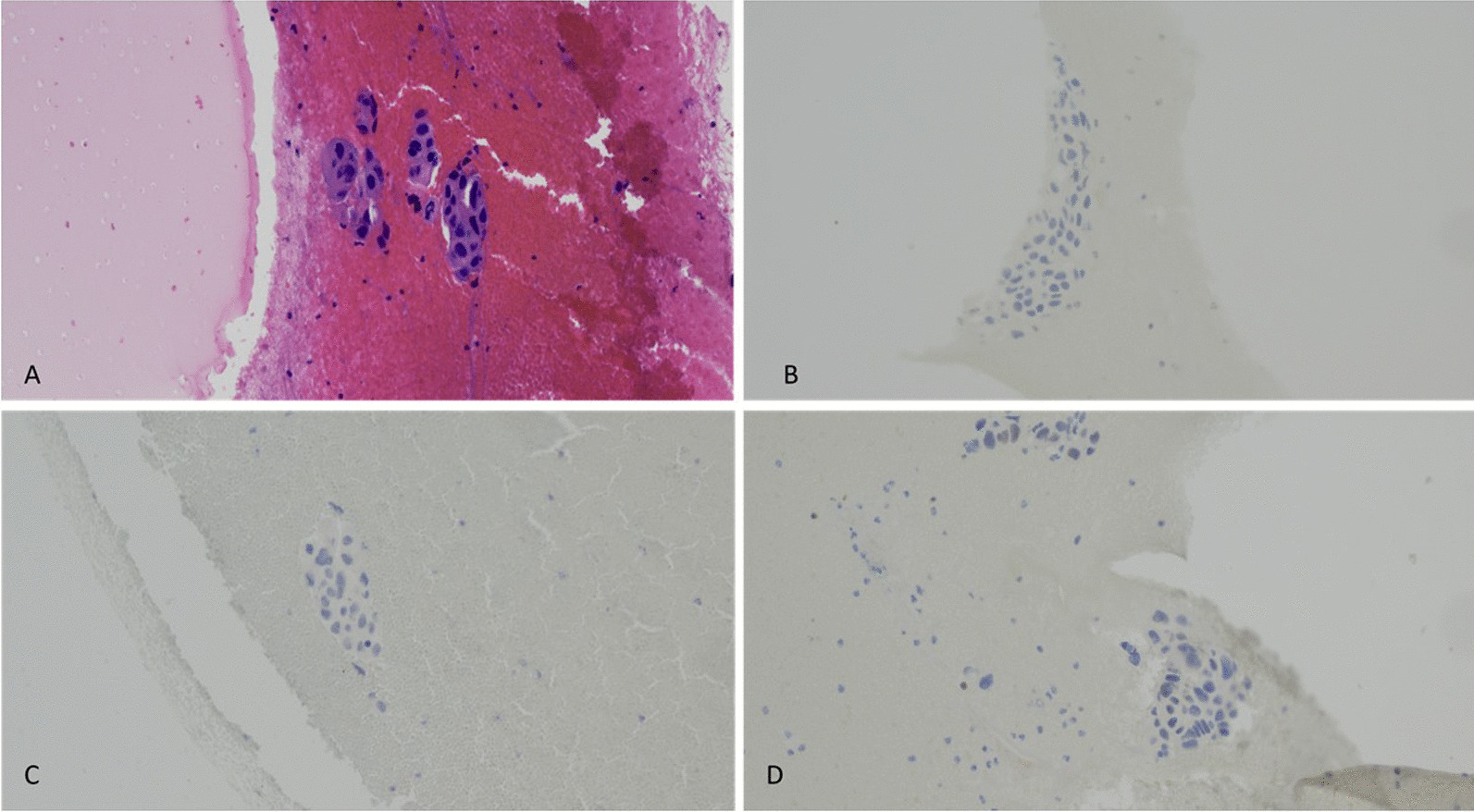
Left subpectoral mass. **A** Hematoxylin and Eosin (H&E) ×40 image of the fine needle aspiration of the left subpectoral mass involving poorly differentiated metastatic ductal carcinoma. **B** Negative estrogen receptor (ER) immunohistochemistry staining of tumor cells. **C** Negative progesterone receptor (PR) immunohistochemistry staining of tumor cells. **D** Negative Her2Neu immunohistochemistry staining of tumor cells

In 2018, at the age of 64 years, while still on anastrozole, she presented with acute left back pain. Images showed recurrent disease with a new left subpectoral mass and left scapular lesion. A biopsy of the mass showed a poorly differentiated carcinoma consistent with ductal carcinoma of mammary origin expressing AE1-3, GATA3, and mammaglobin. However, this time the tumor was ER negative, PR negative, and HER2 negative (FISH copy number 2.1 and ratio 1.4) (Fig. 1A and Fig. 2). Circulating tumor DNA (ctDNA) showed several mutations: *ERBB2* (HER2) S310F of 9.3%, *TP53* Q104 of 0.1%, *TP53* H178fs of 3.7% (Fig. 1 B).

A biopsy of the left scapular lesion showed metastatic carcinoma consistent with breast primary. The bone biopsy was ER negative, PR negative, and HER2 2+, with FISH inconclusive due to technical failure (Fig. 1C). Androgen receptor positive (5–10% weak), GATA3 positive, PD-L1 positive.

She started zoledronic acid and atezolizumab with nab-paclitaxel because of a positive PD-L1 assay and had a partial response by June 2019 (Fig. 1D and E). By September 2019, she showed a mixed response in her follow-up images, with improvement in her left subpectoral lymph node and the lesion in the left coracoid process, but a new lesion at T1 (Fig. 1F). The ctDNA at that time showed *ERBB2* (HER2) S310F 3.8%, *TP53* H178fs 1.9%, and *CDH1* R87fs 0.1% (Fig. 1G).

A repeat positron emission tomography–computed tomography (PET–CT) scan showed improvement of the subpectoral lesion and stable bone metastasis by November 2019 (Fig. 1H). She additionally received stereotactic body radiation therapy (SBRT) to the left coracoid process and the T1 vertebral body to consolidate the therapeutic response (Fig. 1I). The ctDNA done in May 2020 showed *ERBB2* (HER2) S310F 0.5% and *TP53* H178fs 0.4% (Fig. 1 J).

**Fig. 3:**
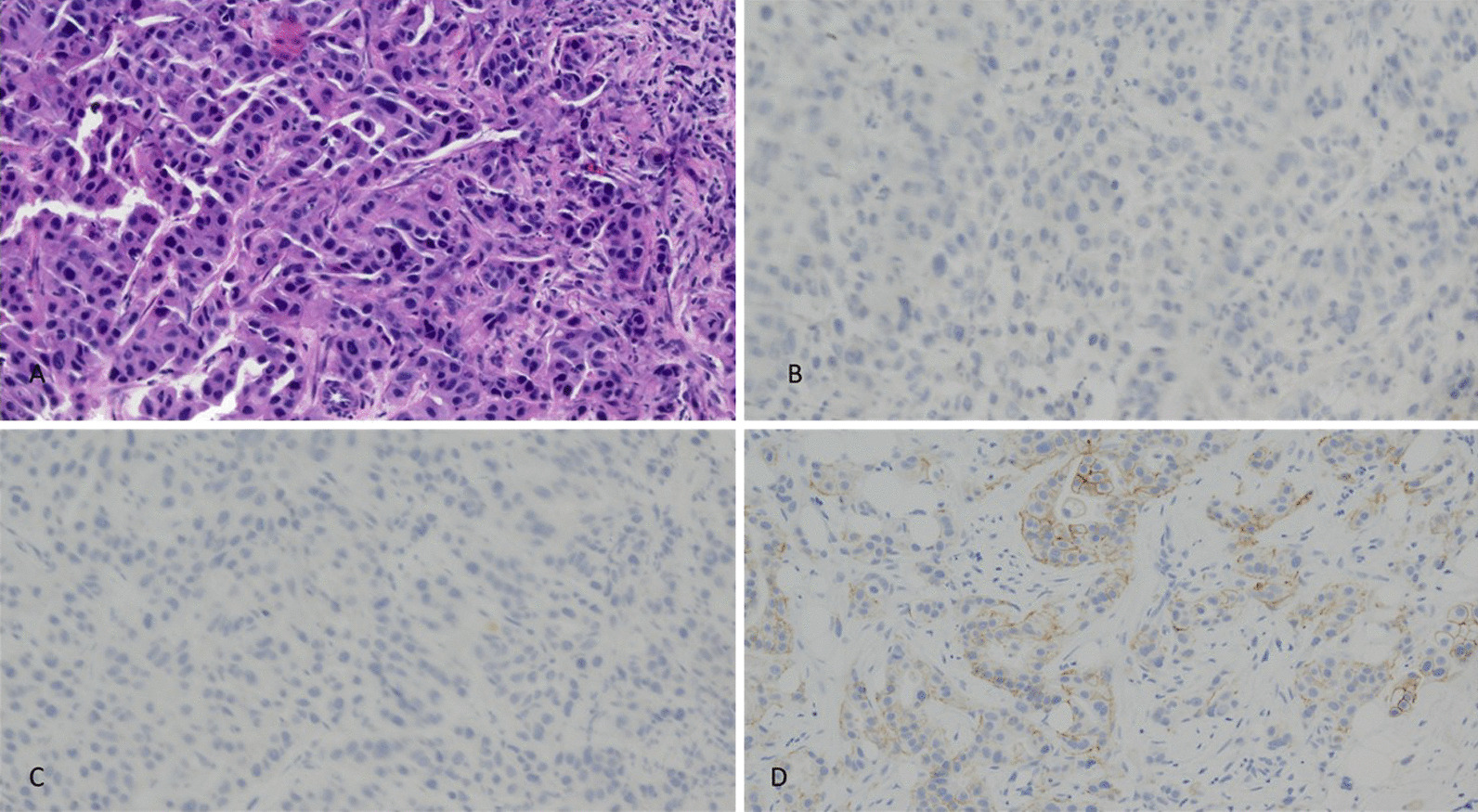
Liver mass. **A** Hematoxylin and Eosin (H&E) ×40 image of the needle core biopsy of the liver mass involved by cytologically similar, poorly differentiated metastatic ductal carcinoma. **B** Negative estrogen receptor (ER) immunohistochemistry staining of tumor cells. **C** Negative progesterone receptor (PR) immunohistochemistry staining of tumor cells. **D** Equivocal (2+) Her2Neu immunohistochemistry staining of tumor cells

By August 2020, she had two small liver lesions. A fine needle aspiration and core needle biopsy of the liver showed an ER negative, PR negative, HER2 2+ by immunohistochemistry (IHC) and positive by FISH group 3 by ASCO CAP 2018 (copy number 7.9 and ratio 1.61) tumor (Fig. 1K and Fig. 3) [10]. This tumor was also AR positive 1%, PD-L1 SP142 positive 2%, PTEN positive 90%, CK7 positive, GATA3 positive, and mammaglobin positive. She had localized treatment by irreversible electroporation (IRE) of the two small separate hepatic lesions to control her disease (Fig. 1L).

The PET–CT done in October 2020 showed that she had overall disease progression in the liver and bone (Fig. 1M). Therefore, her therapy was switched to trastuzumab, capecitabine, and tucatinib (Fig. 1N). A follow-up PET–CT showed a complete response of measurable disease with non-PET fluorodeoxyglucose (FDG)-avid residual lesions viscerally and in bone by January 2021, with sclerotic bone lesions still seen on scan (Fig. 1O). The ctDNA did not detect any somatic alterations in her blood that correlated to the response seen on the PET-CT (Fig. 1P). Her PET–CT done in November 2021 still showed no evidence of active disease (Fig. 1Q).

## Conclusion/Discussion

Receptor conversions are known to occur during the treatment of MBC, but multiple conversions are not well documented. A meta-analysis by Schrijver *et al*. found that receptor conversions of ER, PR, and HER2 from positive to negative were 22.5%, 49.4%, and 21.3%, respectively, while receptor conversions from negative to positive were 21.5%, 15.9%, and 9.5% [8]. This shows that HER2 conversions, especially from negative to positive, are relatively rare when compared with the hormone receptor (HR) status. Meng *et al*. performed a retrospective review looking at the ER and PR receptor status of primary tumors and metastatic sites [11]. They found that a conversion occurred in 26.3% of patients [11]. Some patients had additional biopsies and they were able to reassess for re-conversions. They reported that ER and PR had a re-conversion in 26.8% and 35.4% of cases, respectively, but there was no mention of HER2 re-conversions [11].

A study by Hoefnagel *et al*. showed that discordance of diverse metastatic sites within the same patient for ER was 7.3% or 10.9% (using, respectively, a 10% or 1% threshold for positivity), for PR it was 29.1% or 30.9%, and for HER2 it was 3.6% on IHC but 0% for gene amplification [12]. Thus in 10.9–14.5% of the patients, this discordance might have clinical relevance indicating a potential need to change treatment [12]. Additionally, this study demonstrated that there was minimal discrepancy within metastatic lesions (for example, heterogeneity) for HER2, making this case that much more uniquely valuable. Since receptor conversions can occur in the course of disease progression from the primary tumor to subsequent metastatic sites, the National Comprehensive Cancer Network (NCCN) guidelines recommend a biopsy of the metastatic site at presentation [13]. During the course of our patient’s treatment, we biopsied at presentation and at multiple progressions to reassess receptor status and the presence or absence of actionable mutations to optimize treatment.

Receptor conversions can change the patient’s prognosis. While HER2 conversions from positive to negative are associated with a poor prognosis, HER2 conversions from negative to positive are associated with better outcomes [8, 9]. Circulating tumor DNA (ctDNA) can be helpful in certain circumstances, but it will not detect a change in HR status and is not as sensitive as a tissue biopsy in detecting HER2 amplification [14]. Tissue biopsies are still the gold standard for molecular analysis and have better sensitivity for assessment of amplification [15].

The receptor status of the patient is important in providing the clinician with the most accurate information for choosing appropriate treatment. If the receptor status changes, then the patient may be receiving ineffective treatment and additional toxicity without therapeutic benefit. The question then becomes how often or when should one be biopsying the patient’s lesions to potentially change therapeutic treatment? In this case, we decided to track her ctDNA along with obtaining multiple metastatic biopsies at the time of progression. We tracked her metastatic response to treatment through imaging examinations, and when it was evident that she had disease progression, we biopsied her lesions and found, once again, she had a receptor conversion.

Circulating tumor DNA offers fast and accurate genotyping in patients with MBC, which helps in the selection of therapies in patients with actionable mutations [16]. The use of ctDNA was interesting in that she had an *ERBB2* mutation that later disappeared. Nevertheless, it missed the HER2 amplification. This shows that even though ctDNA can sometimes be useful to track how well a patient is responding to treatment, it can still miss some changes in the tumor. A tissue biopsy of the metastatic lesion, when appropriate, is the best test to assess changes. Given the invasive nature of a tissue biopsy, it should be considered when clinically there is some suggestion of a differing disease process, as in our case. As previously shown, our patient’s ctDNA was going down while she developed a new liver lesion.

A study by Nayar *et al*. found that HER2 mutations and ER mutations are mutually exclusive, suggesting a separate mechanism of acquired resistance for each one [17]. Moreover, HER2 mutations are relatively rare, accounting for 1.6% of primary BC’s [17]. For women with HER2 mutations, the SUMMIT basket trial demonstrated encouraging clinical results for the use of neratinib alone or in combination with fulvestrant [18].

Metastatic BC shows high mutational burden and clonal diversity when compared with early BC [19]. This could be due to selective spread across metastatic sites of positive and negative clones from the primary lesion, clonal selection during disease progression possibly due to therapeutic treatment, genomic evolution during the course of metastasis, biological drift, or small undetectable subclones in primary tumors that only become evident once metastasis has occurred [12, 20]. It is possible that this is what happened to our patient. She could have had heterogeneity within the initial MBC tumor that was not originally detectable. Her original BC diagnosis was HR+ HER2− then her initial soft-tissue MBC lesion biopsy was HR− and HER2− by FISH. At the time of progression, her liver was biopsied and the tumor was HER2+ by FISH.

Molecular imaging could help target which lesions to biopsy. In a meta-analysis by Kurland *et al*. they recommend the integration of PET with 16α-18F-fluoro-17β-estradiol (F-FES) as an image biomarker tracer to identify areas of ER metastatic lesions [21]. Hopefully in the future, additional tracers are integrated to determine areas of marker diversities to minimize random metastatic biopsy selection.

This report describes a case where multiple conversions occurred. It is therefore extremely important to not only perform a biopsy at the time of metastatic diagnosis, but also during the course of metastatic treatment.

## Data Availability

Not applicable.

## Abbreviations

BC: Breast cancer
ER: Estrogen receptor
HER2: Human epidermal growth factor receptor 2
MBC: Metastatic breast cancer
PR: Progesterone receptor
FISH: Fluorescence in situ hybridization
TAC: Docetaxel, doxorubicin, and cyclophosphamide
ctDNA: Circulating tumor DNA
PET–CT: Positron emission tomography–computed tomography
SBRT: Stereotactic body radiation therapy
IHC: Immunohistochemistry
IRE: Irreversible electroporation
FDG: Fluorodeoxyglucose
HR: Hormone receptor
NCCN: National comprehensive cancer network
F-FES: F-fluoroestradiol
